# Synchronous right colon adenocarcinoma and sigmoid endometriosis mimicking a second primary carcinoma: a case report

**DOI:** 10.3389/fsurg.2026.1923049

**Published:** 2026-08-13

**Authors:** Ke Yang, Ping Li, Xuan Long

**Affiliations:** Department of General Surgery, Mianyang Central Hospital, School of Medicine, University of Electronic Science and Technology of China, Mianyang, Sichuan, China

**Keywords:** case report, colorectal cancer, endometriosis, frozen section, synchronous lesions

## Abstract

Intestinal endometriosis is a benign gynecological condition that frequently involves the rectosigmoid colon. Its synchronous occurrence with colorectal cancer is exceptionally rare and diagnostically challenging, particularly when mimicking a second primary malignancy. We report a case of a 48-year-old woman presenting with altered bowel habits. Colonoscopy revealed synchronous lesions: an adenocarcinoma at the hepatic flexure and a suspicious mass in the sigmoid colon. Repeated biopsies of the sigmoid lesion were inconclusive, ranging from dysplasia to chronic inflammation, raising concern for a second primary tumor. The patient underwent right hemicolectomy with partial sigmoidectomy. Intraoperative frozen section unexpectedly identified endometriosis, subsequently confirmed by definitive pathology and immunohistochemistry. At 12-month follow-up, the patient remained well with no evidence of recurrence.

## Introduction

Intestinal endometriosis predominantly affects the rectosigmoid colon in 3.8%–37% of women, causing chronic pelvic pain, dysmenorrhea, dyspareunia, and cyclical bowel changes that impair quality of life (1–3). Its clinical, endoscopic, and imaging features closely resemble colorectal cancer, yet preoperative pathological confirmation is rarely achieved because lesions reside in the submucosa and standard biopsies yield only nonspecific inflammation (4, 5). Consequently, definitive diagnosis is usually established postoperatively via paraffin pathology and immunohistochemistry (6).

Although numerous cases of intestinal endometriosis have been reported in the literature, many were misdiagnosed as colorectal cancer and underwent unnecessary surgical resection (7–9). However, the specific scenario of right colon cancer with synchronous sigmoid endometriosis, as presented in our case, has not been previously reported. We report such a case, highlighting the diagnostic pitfalls and the critical roles of intraoperative frozen section and multidisciplinary collaboration in guiding surgical strategy.

## Case presentation

A 48-year-old woman was admitted to our hospital with a 6-month history of altered bowel habits, characterized by frequent (3–4 times daily), loose, and mucopurulent stools. The timeline of the main diagnostic and therapeutic events during surgical care is summarized in Table 1. She had previously undergone appendectomy, hemorrhoidectomy, and excision of breast nodules. She had no smoking or significant alcohol history, with regular menses and no dysmenorrhea. Family history was negative for hereditary or major infectious diseases. Physical examination revealed no abnormal findings on abdominal palpation, and digital rectal examination was unremarkable. Laboratory tests, including complete blood count, liver and renal function, electrolytes, coagulation profile, and tumor markers (CEA 2.24 ng/mL, CA125 32.35 U/mL, CA19-9 36.8 U/mL), were all within normal limits. The patient's hemoglobin was 123 g/L and serum albumin was 45 g/L, which were within normal ranges—likely reflecting the early stage of the tumor (as confirmed by postoperative pathology) and the absence of significant bleeding or malnutrition.

**Table 1:** Timeline of the main diagnostic and therapeutic events during surgical care.

Timeline of the case	
Date	Event
17-Dec-24	First outpatient visit for altered bowel habits
20-Dec-24	Initial colonoscopy + biopsy: ascending colon adenocarcinoma; sigmoid lesion with dysplasia
23-Dec-24	Repeat biopsy: sigmoid lesion showed only chronic inflammation
24-Dec-24	Preoperative CT and MRI scan
10-Jan-25	Admission of patient to surgical department
14-Jan-25	Laparoscopic surgery + intraoperative colonoscopy + frozen section → endometriosis diagnosed; right hemicolectomy + sigmoid resection performed
27-Jan-25	Discharged, uneventful recovery
17-Jan-26	12-month follow-up: no recurrence

**Figure 1:**
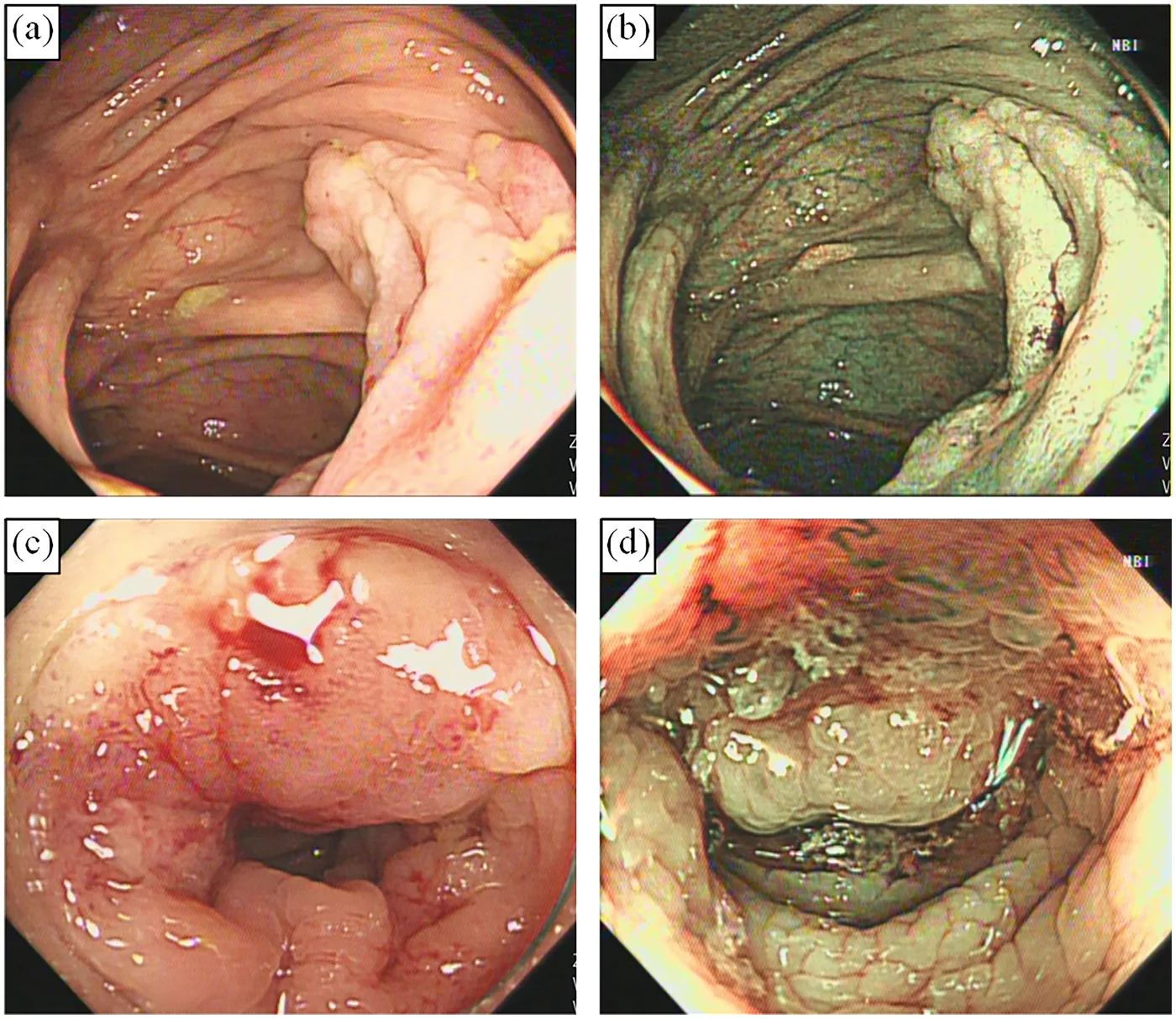
Colonoscopic findings. **(a,b)** Adenocarcinoma at the hepatic flexure; **(c,d)** Lesion in the sigmoid colon.

**Figure 2:**
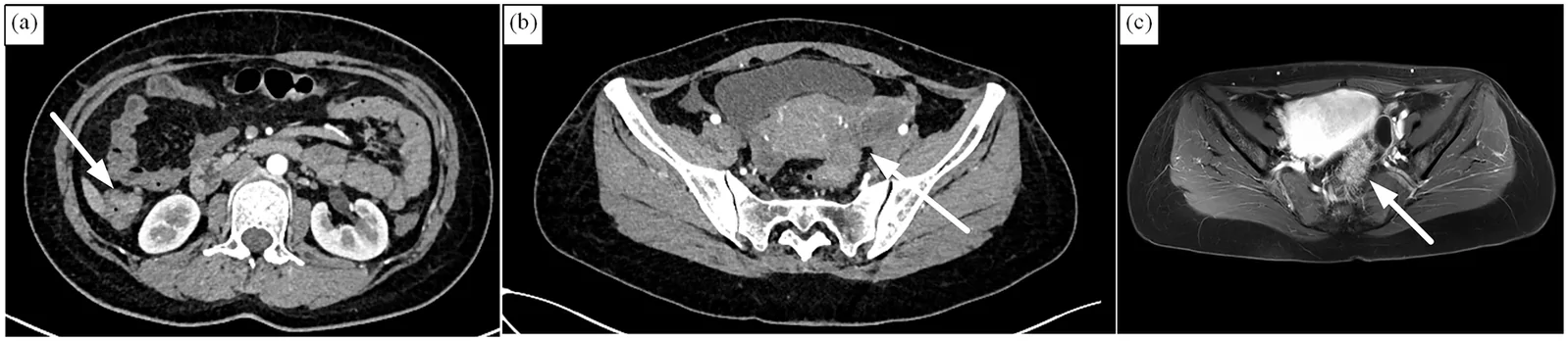
Imaging findings. **(a)** Contrast-enhanced CT of the ascending colon showing mild wall thickening at the hepatic flexure (white arrow). **(b)** Contrast-enhanced CT of the sigmoid colon showing circumferential wall thickening (white arrow). **(c)** Contrast-enhanced MRI demonstrating similar findings in the sigmoid colon with ill-defined borders (white arrow).

Colonoscopy revealed two synchronous lesions: a laterally spreading tumor at the hepatic flexure of the ascending colon, and a semi-circumferential mass located 17–19 cm from the anal verge in the sigmoid colon, occupying approximately half of the lumen (Figure 1). Histopathological examination of biopsies from the ascending colon lesion confirmed intramucosal adenocarcinoma. Biopsies from the sigmoid lesion were inconclusive—initially showing glandular dysplasia, raising suspicion for malignancy. A repeat sigmoidoscopy with targeted biopsy was subsequently performed, which demonstrated only moderate chronic inflammation with erosion. Contrast-enhanced CT of the abdomen and pelvis demonstrated mild and heterogeneously enhancing wall thickening of the ascending colon. The distal sigmoid colon showed approximately 1.0 cm of wall thickening with ill-defined borders and homogeneous enhancement, the nature of which was indeterminate. The uterus was enlarged with an irregular contour, suggestive of adenomyosis. Pelvic contrast-enhanced MRI confirmed these findings (Figure 2).

**Figure 3:**
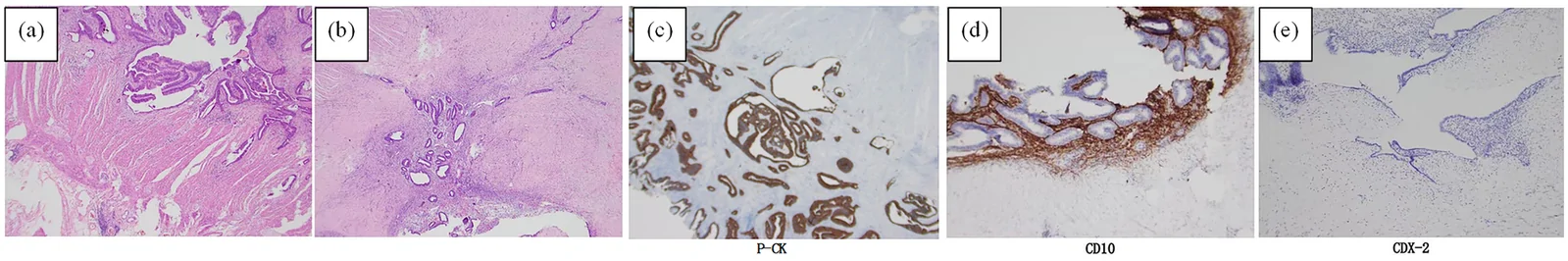
Pathological and immunohistochemical findings. **(a)** Ascending colon adenocarcinoma (H&E). **(b)** Sigmoid endometriosis (H&E). **(c)** P-CK staining (ascending colon adenocarcinoma). **(d)** CD10 staining (sigmoid lesion). **(e)** CDX-2 staining (sigmoid lesion).

Following preoperative multidisciplinary discussion involving gastrointestinal surgery, gynecology, and oncology, the patient was diagnosed with right colon cancer with a synchronous sigmoid lesion of indeterminate nature, without evidence of distant metastasis. Laparoscopic exploration was subsequently performed. Intraoperative findings included a 2 cm × 2 cm exophytic mass at the hepatic flexure and dense adhesion of the rectosigmoid junction to the cervix, forming a firm, ill-defined mass. Intraoperative colonoscopy was employed to delineate the distal tumor margins. Frozen section analysis of the sigmoid lesion unexpectedly returned a diagnosis of endometriosis, and a gynecologist was consulted intraoperatively. The definitive procedure consisted of right hemicolectomy (*en bloc* resection of the terminal ileum, cecum, ascending colon, and right branch of the middle colic vessels with regional lymphadenectomy) and segmental resection of the sigmoid colon with a 5-cm proximal and distal margin, followed by primary anastomosis, as well as uterine curettage. Final pathological examination confirmed a moderately differentiated adenocarcinoma of the ascending colon (pT2N0M0, Stage I), endometriosis of the sigmoid colon, and proliferative-phase endometrium in the uterine curettage specimen. No further resection of the adnexa or uterus was performed. Immunohistochemistry supported both diagnoses: the ascending colon adenocarcinoma showed diffuse positivity for P-CK, confirming its epithelial origin; the sigmoid lesion showed strong positivity for ER (90%), PR (90%), CD10, CK7, and PAX-2, with negative staining for CDX-2, CK20, and SATB2, confirming endometrial origin (Figure 3). The patient's postoperative recovery was uneventful, and she was discharged on the 13th postoperative day. At 1-year follow-up, she remained well with no evidence of recurrence.

## Discussion

Endometriosis is characterized by functioning endometrial tissue beyond the uterine cavity, affecting up to 15% of reproductive-aged women, with gastrointestinal involvement occurring in as many as 37% of cases (10). Intestinal endometriosis may present with chronic cyclic abdominal pain and altered bowel habits, often mimicking irritable bowel syndrome and leading to years of misdiagnosis (11). Historical clues such as dyspareunia, unexplained infertility, dysmenorrhea, known endometriosis, and perimenstrual symptom exacerbation may aid diagnosis, though physical examination is usually normal or reveals only nonspecific findings. Establishing a definitive diagnosis of intestinal endometriosis is particularly elusive, because the lesions typically spare the mucosa and reside in the deeper layers of the bowel wall (12, 13). Consequently, mucosal biopsies obtained via standard colonoscopy often reveal only normal mucosa or non-specific inflammatory changes, making the procedure more valuable for ruling out other pathologies than for establishing a diagnosis of intestinal endometriosis. Even in the presence of overt endoscopic abnormalities—such as stricture formation or submucosal protrusions—histopathological examination frequently shows no diagnostic features, resulting in a substantial rate of false-negative findings (14).

Various imaging modalities have been employed to evaluate intestinal endometriosis. Abdominal CT or MRI may demonstrate bowel wall thickening, with MRI offering superior delineation of endometriomas and multifocal lesions (15). Endoscopic ultrasound (EUS) can further characterize the layers of bowel wall involvement, the length of affected segment, distance from the anal verge, and the presence of reactive lymph nodes or extracolonic disease (15, 16). Despite these imaging advances, a definitive diagnosis of endometriosis without laparoscopic confirmation remains difficult to establish.

The diagnostic challenge posed by intestinal endometriosis is well illustrated by previously reported cases. In 2018, Masatsugu Ishii et al. reported a case series of seven patients with intestinal endometriosis who were preoperatively mistaken for colorectal cancer and underwent surgical resection (8). Other reports have described rectal endometriosis misdiagnosed as adenocarcinoma or presenting with bowel obstruction (7, 9). Beyond the pelvis, endometriosis can also involve distant sites, further complicating the differential diagnosis—one case described a rectal cancer patient with a synchronous mediastinal mass that, after resection, proved to be endometrial carcinoma arising from mediastinal endometriosis (17). This case further illustrates the ability of endometriosis to mimic metastatic disease and occasionally undergo malignant transformation. However, none of these reports described a synchronous endometriotic lesion in a patient with established right colon cancer, highlighting the distinctiveness of our presentation.

Given these diagnostic pitfalls, a thorough understanding of the clinical behavior of endometriosis is essential for appropriate surgical management. Endometriosis is generally considered a benign condition, yet accumulating evidence suggests a potential link with certain malignancies. Moreover, patients with endometriosis appear to be at higher risk for several chronic diseases, and malignancy is frequently suspected in this population, particularly given that gynecologic cancers may coexist with endometriosis (18). In light of these clinical concerns, timely and accurate surgical management becomes particularly important in cases of suspected intestinal involvement. Once surgical intervention is indicated for intestinal endometriosis, resection remains the mainstay of treatment. The operative objectives are early recognition, complete excision of ectopic endometrial tissue, and alleviation of symptoms. Laparoscopic approaches for colorectal conditions have evolved substantially in recent years and have proven valuable not only for therapy but also for distinguishing endometriotic lesions from colorectal malignancies (19). The safety and feasibility of laparoscopic colorectal resection for endometriosis have been corroborated by multiple investigations, including a prospective randomized study conducted by Darai and colleagues (20).

In China, the incidence and mortality of colorectal cancer have steadily increased, with approximately 520,000 new cases and 240,000 deaths reported in 2022 (21). The prevalence of synchronous colorectal cancer ranges from 1.1% to 8.1% (22). In this context, the presence of a confirmed malignancy elsewhere in the colon creates a cognitive bias toward malignancy, making the consideration of benign etiologies exceedingly challenging. Given the limitations of preoperative biopsies and imaging, we turned to intraoperative tools to guide our surgical strategy. Frozen section evaluation plays a crucial role in this setting, offering real-time diagnostic insight that influences surgical decisions (23). In our case, despite the indeterminate nature of the sigmoid lesion, the confirmed diagnosis of right colon adenocarcinoma without distant metastasis prompted laparoscopic exploration. Intraoperative frozen section subsequently confirmed sigmoid endometriosis, and concurrent colonoscopy precisely delineated the distal margin, ensuring negative margins. Together, these intraoperative tools transformed a planned radical resection into a more conservative, organ-sparing procedure that balanced oncologic cure with functional preservation. This team-based approach enabled us to achieve the best possible diagnostic and therapeutic outcome for the patient.

## Conclusion

For female patients presenting with multiple colorectal lesions, the possibility of intestinal endometriosis should be carefully considered, particularly when preoperative workup yields inconclusive results. During surgery, effective communication between the operating surgeon and the histopathologist within the framework of interdisciplinary cooperation is essential to achieve the best possible patient outcomes.

## Data Availability

The original contributions presented in the study are included in the article/Supplementary Material, further inquiries can be directed to the corresponding author.
